# Patient perspectives of pain and function after knee replacement: a systematic review and meta-synthesis of qualitative studies

**DOI:** 10.1097/PR9.0000000000001006

**Published:** 2022-05-09

**Authors:** Carrie E.V. Taylor, Carolyn M. Murray, Tasha R. Stanton

**Affiliations:** a IIMPACT in Health, Allied Health & Human Performance Academic Unit, University of South Australia, Adelaide, Australia; b International Centre for Allied Health Evidence, Allied Health & Human Performance Academic Unit, University of South Australia, Adelaide, Australia

**Keywords:** Total knee replacement, Pain, Function, Recovery norms, Mental health, Communication, Qualitative

## Abstract

**Introduction:**

Joint replacement surgery typically results in good clinical outcome, although some people experience suboptimal pain relief and functional improvement. Predicting surgical outcome is difficult.

**Objectives:**

There is merit in better understanding patients' perspectives of pain and function to identify avoidable problems perceived to contribute to their outcome, to inform prognostic expectations, and to identify potential cointerventions to sit alongside surgery that might mitigate pain/functional problems. Here, we aimed to synthesise the available literature exploring perspectives of people with knee osteoarthritis about their pain and function following joint replacement.

**Methods:**

Six electronic databases and 2 websites were searched. Two independent reviewers completed study inclusion, quality assessment, and data extraction. Data were iteratively synthesised using first-, second-, and third-order analyses.

**Results:**

Twenty-eight studies were included. Four themes were identified; perceptions of pain and function were inseparable. Theme 1 addressed experiences of recovery after surgery, which often differed from expectations. Theme 2 described the challenges of the pain experience and its functional impact, including the difficulty navigating medication use in context of personal beliefs and perceived stigma. Theme 3 articulated the toll of ongoing problems spanning pain–function–mood, necessitating the need to “endure.” Theme 4 encompassed the importance of clinical/social interactions on mood and pain, with reports of concerns dismissed and practical support missing.

**Conclusions:**

Together, these findings show that numerous individual considerations beyond the technical aspects of surgery influence experiences of pain and function. A tailored approach addressing these considerations from the patient perspective could provide a basis for improved success of knee replacement surgery.

## Abstract 2

Supplemental Digital Content is Available in the Text.

Patient perspectives highlight the need for presurgical information about expected recovery trajectories after total knee replacement and enhanced postsurgical follow-up with practical support.

## 1. Introduction

Knee osteoarthritis (OA) is a leading cause of pain and disability in older adults^29,73^ affecting millions world-wide.^18^ Osteoarthritis is the most common reason for total knee replacement (TKR)^2^ with clinical guidelines recommending consideration of TKR after nonsurgical care is offered/trialled.^8,52,62^ Yearly rates of TKRs continue to increase^1,2,53^ and with up to 68% of general practitioners referring patients directly to orthopaedic surgeons (ie, by-passing recommended conservative care prior to surgery), TKR may occur prematurely in some individuals,^8^ potentially contributing to suboptimal outcomes.

People with knee OA expect benefits from surgery such as reduction or elimination of pain and restoration of function. Quantitative methods to evaluate TKR outcomes often assess surgical success, including joint survivorship^3^ and prosthetic alignment (via imaging).^72^ While patients' self-reports of pain and function after TKR are mainly positive,^34^ 10 to 34% have unfavourable long-term pain outcomes following TKR, 15% report moderate or severe pain (2–5 years post-TKR),^6^ and many report ongoing functional difficulties.^77^ Residual pain and functional limitations raise issues about what constitutes surgical success after TKR^31^ and create challenges for optimal management because it is difficult to predict who will experience suboptimal pain and functional outcomes postsurgery.^55^ Comprehensive understanding of TKR outcomes and experiences from those who have undergone the procedure is needed. Specifically, eliciting in-depth patient perspectives about TKR via open qualitative inquiry provides opportunity to capture new information not available through the assessment via traditional self-report measures.

Thus, the aim of this research was to conduct a meta-synthesis of the available qualitative literature to understand the patient perspective of pain and function following TKR. Understanding patients' experiences of pain and function after TKR, both positive and negative, provides a body of contextual evidence that people undergoing TKR can relate to and that health professionals can learn from. Exploring the perspectives of people undergoing TKR also provides greater depth and quality of information concerning likely recovery trajectories, thus informing prognostic expectations for future surgical candidates. Importantly, information attained about challenges and problems experienced by those undergoing TKR can provide a basis for the development of additional conservative interventions that may sit alongside medical interventions to mitigate pain and functional problems. Such information can also inform the weighting of surgical risks against potential benefits (or non-benefits) when making the decision to undergo TKR.

## 2. Method

### 2.1. Study design, registration, and reporting

This review used a qualitative thematic synthesis design,^65^ and the protocol was prospectively registered on PROSPERO (CRD42020190075; https://bit.ly/3gRwMlK). The Enhancing Transparency in Reporting the Synthesis of Qualitative Research (ENTREQ)^66^ approach guided reporting (Supplementary File 1, available at http://links.lww.com/PR9/A159).

### 2.2. Data sources and search strategy

Database search strategies for Embase, Emcare, Cochrane Library, Medline, ProQuest, PsycINFO, and Scopus were developed in conjunction with an academic librarian and run from database inception to May 18, 2021. Keywords relating to knee replacement, qualitative research, and pain or function were used as well as subject headings specific to each database (Supplementary File 2, available at http://links.lww.com/PR9/A159). Searches in Trove and in Google Scholar were undertaken using keyword combinations to identify studies not indexed in bibliographic databases, with the first 100 results of each search included. The reference lists of included articles were also hand searched.

### 2.3 Eligibility criteria and study selection

SPIDER criteria^15^ were used to define the study question and to guide study eligibility (Table 1).

**Table 1:** SPIDER criteria and eligibility criteria for study selection.

SPIDER criteria	Eligibility criteria and rationale
Sample population (S)	Community-based older adults (aged 60 y and above) with knee OA Community-dwelling older adults were selected to avoid the significant comorbidities and functional mobility difficulties experienced by those in residential care that may influence surgical outcome and thus perspectives of pain and function.^33,37^ A minimum age of 60 y was chosen to target the typical population undergoing TKR for knee OA. People younger than 60 y needing TKR often require surgery for trauma-induced OA or for rheumatoid arthritis, both of which may result in different clinical trajectories.^50,75^ Evidence also suggests that younger adults may have higher expectations of TKR outcome and pain and functional recovery than older adults after surgery.^76^ In addition, perceptions of pain and function may be influenced by reduced activity levels in older adults following TKR as compared with younger adults,^39^ and higher rates of surgical complications in older adults.^25^ When studies recruited a sample that included those younger than 60 y, studies were eligible for inclusion if (1) data specific to those aged 60 y and older was able to be extracted or (2) if 75% or greater of the sample were older than 60 y of age (assessed using sample means/standard deviations), in which case all data were extracted.
Phenomenon of interest (PI)	Have undergone TKR If the recruited study population involved a mix of people with various lower limb joint replacements (eg, hip and knee), the study was eligible for inclusion if 75% or greater of the sample underwent a TKR (full data extracted) or if the TKR participants could be specifically identified (only TKR data extracted); otherwise, these studies were ineligible.
Research designs (D)	Qualitative methodology and data collection methods (ie, interview, focus groups)
Evaluation (E)	Perspectives of pain and/or function following TKR surgery in the sample population
Research type (R)	Qualitative or mixed methods studies published after 2002 Studies published after 2002 were deemed most relevant to capture perspectives about modern surgical techniques and allow potential comparison with outcome measure data available through international joint replacement registries in the United Kingdom, Australia, Scandinavia, and United States.^24^ Only studies written in English were included because this review was unfunded, and resources were not available to facilitate the necessary in-depth translation of non-English qualitative studies.

Searches were run from database inception to May 2021, but following consultation with surgical colleagues, the decision was made to focus on studies published from 2002 onwards to capture patient experiences relevant to modern surgical technique and pre- or postoperative care procedures. Limiting the studies to the past ∼20 years also allows potential comparison of patient perspectives about outcomes from TKR to surgical outcomes reported in existing joint replacement outcome data registries.^1,2,21,24,53^

### 2.4. Data handling and study inclusion

Search results were imported to EndNoteX9 (www.endnote.com; Clarivate Analytics, Philadelphia, PA), and duplicates removed. Results were then exported to Covidence (www.covidence.org; Veritas Health Innovation, Melbourne, Australia) for further automatic removal of duplicates and for screening. Two independent reviewers (C.T. and C.M.) completed title and abstract screening to remove obviously irrelevant studies, followed by formal full-text screening applying the full eligibility criteria. When conflicts arose, these were resolved by discussion and when needed, consultated with a third independent reviewer (T.S.).

### 2.5. Quality assessment

The Critical Appraisal Skills Program (CASP) Qualitative Check list^17^ was used to assess study quality. A scoring system was used^44^ to provide numerical ratings for items 1 to 9 (Supplementary File 3, available at http://links.lww.com/PR9/A159): items were scored 3 when sufficient explanation of the criterion was provided (highest score), scored 2 when the criterion was addressed but without full explanation, or scored 1 when offering little to no explanation of the criterion (lowest score). Total scores ranged from 9 to 27, with higher scores indicating higher quality. Item 10 (Utility) was not scored because the transferability of the research depends on individual practice contexts. Two reviewers (C.T. and C.M.) independently scored each study. The scores were then reviewed and compared by the lead author, and where discrepancies occurred, consensus was reached through discussion to produce the final score. Percentage agreement and prevalence- and bias-adjusted kappa (PABAK) statistics were used to evaluate interrater reliability, considering ratings for items 1 to 9.^19,42^

### 2.6. Data extraction

Two independent reviewers (C.T. and C.M.) used customised, piloted forms to extract the following data from each study: Study title; year of publication; country; study aim; study design; data collection methods; sample characteristics (number of total participants in study, number of participants with TKR over 60 years, number of males/females, mean age, age range); summary of findings relating to TKR participants; and patient perspectives of pain and function taken from study results and discussion, including participant quotations and identified themes. Conflicts were resolved through discussion and additional consultation with the third reviewer (T.S.) as needed.

### 2.7. Data analysis

Best practice analytical techniques for qualitative meta-synthesis, involving a 3-stage process of analysis, were undertaken.^70^ The first stage was undertaken by the first author, identifying codes “line by line”^70^ for each element of data extracted about perceptions of pain and function (eg, participant quotes, descriptive phrases, and sentences taken from the included studies). The individual data items along with their initial code and the number of the study it had been extracted from were printed on separate pieces of paper. The second stage involved manually sorting the coded data pieces into categories, via group analysis with all 3 authors. The third stage involved inductive categorisations to reduce and consolidate the data into “descriptive themes.”^70^ Further group discussions occurred over multiple meetings to generate “analytical themes.”^70^ Once analytic themes were constructed, further analysis by the first author involved writing up themes, iteratively reorganising themes and rechecking for context against the original sources before all authors reached consensus about the final themes and subthemes.^54,70^

### 2.8. Rigour

To ensure study rigour, numerous steps were undertaken. First, during both stages of screening, the researchers were blinded to each other's decisions to ensure low risk of bias for inclusion decisions. Blinding was achieved via the use of Covidence, whereby researchers were unable to see the screening decisions of others until all studies within the review stage had been rated by 2 reviewers. Second, using multiple researchers ensured varied perspectives during qualitative synthesis, reducing risk of biased interpretation. Third, management of investigator bias was minimised through formal reflection (eg, reflexivity), documentation and declaration of assumptions (eg, bracketing), and having multiple data analysts engaging in discussion to reach consensus about results. Finally, clear reporting using the ENTREQ guide^71^ and documentation of an analysis audit trail supports replicability.

## 3. Results

**Figure 1.**
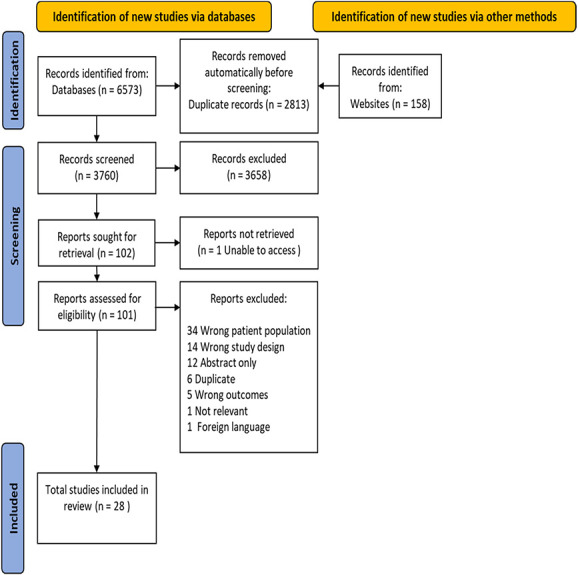
PRISMA flowchart of study screening and selection.

Of a total of 6728 studies identified by the search strategy, 28 studies met the eligibility criteria and were included in the review (see Supplementary File 4, available at http://links.lww.com/PR9/A159). A PRISMA flow chart, including reasons for study exclusion, is depicted in Figure 1.

### 3.1. Characteristics of included studies

Twenty-eight studies^5,9,10,12,16,23,26,32,35,36,40,41^ from 11 countries,^45,47–49,51,57,60^ spanning 4 regions,^64–67,69,74,77,78,82^ were included (Table 2). The oldest study was published in 2004 and the most recent in 2020. They contained approximately 433 participants, of which approximately 54.3% were female and 45.7% male (estimated due to reporting differences between studies).

**Table 2:** Study characteristics.

Study and country	Study aim	Study design/methods	Participant demographics	Summary of findings relating to pain and function
Berg et al.^5^; Sweden	To understand patient experience of fast-track elective total hip replacement and total knee replacement to identify factors influencing recovery and clinical outcome	Interviews 3 mo after surgery Inductive content analysis	N = 24 (11 TKR) Mean age 65 Age range 44–85 >60 = 17 Inclusion = 8 2F/6M	The findings describe 3 distinct stages of fast-track total knee and hip replacement surgery care: pre, during, and postsurgery. All stages indicate the importance of person-centred care, communication, and information provision. The authors suggest that focus on postdischarge care may improve recovery, patient satisfaction, and function.
Bremner^9^ (THESIS) USA Bremner et al.^10^; USA	To understand elderly patient experience of the postoperative period and their medication use	Qualitative descriptive approach Semistructured face to face and telephone interviews	N = 14 (14 TKR) Mean age 74.07 Age range 66–80 >60 = 14 6F/8M	The findings describe the ways participants adapted their pain medication usage to their individual needs. The author suggests that patients need access to more professional advice and guidance about analgesia postdischarge.
Bunzli et al.^12^; Australia	To explore knowledge gaps and misconceptions after total knee replacement surgery	Prestudy recruitment with questionnaire on expectations followed by interviews. Inductive thematic analysis	N = 20 (20 TKR) Mean age* Age range 50–80+ >60 = 19 10F/9M	The findings describe the divergence of what patients expect from total knee replacement surgery when compared with actual experiences of pain and function. They consider that patients have significant gaps in their understanding leading to misconceptions about total knee replacement surgery process and outcomes.
Coutu et al.^16^; Canada	To gain insight into factors influencing sustainable return to work following total knee replacement	Mixed methods with a qualitative descriptive multiple case study design, semistructured interviews, thematic analysis	N = 17 (17 TKR) Mean age 53.2 Age range 42–63 >60 = 5 3F/2M	The findings concentrate on the level of difficulty patients experienced and the reasons why workers returned or did not return to work after total knee replacement.
Engström et al.^23^; Sweden	To describe women's experiences of undergoing total knee joint replacement surgery	Structured interviews Purposive sampling Content analysis	N = 5 (5 TKR) Mean age* Age range 62–84 >60 = 5 5F/0M	The findings describe the periods before, during, and after surgery. After surgery, it appears that patients are happy to have undergone TKR, despite issues around the length of recovery, pain, and challenges in regaining function and that support from health care professionals impacted the patient's experiences. The authors suggest that health care professional support is important across all 3 stages.
Fletcher et al.^26^; UK	To explore the long-term impact and service needs of kneeling difficult after knee replacement	Semistructured telephone interviews Content analysis	N = 56 (56 TKR) Mean age* Median age 75 Age range 71–80 >60 = 56 39F/17M	The findings postoperatively concentrate on the impact of kneeling ability on household activities, leisure activities, and self-care. These were modified with patients adapting to their limitations, patient mood, and support (or lack of it) regarding kneeling restrictions. The authors suggest that there are unmet information needs relating to kneeling.
Harding et al.^32^; Australia	To explore people's beliefs and perspectives about physical activity 6 mo following total hip arthroplasty and total knee replacement	Descriptive interpretative methodology Semistructured interviews Thematic analysis	N = 10 (5 TKR) Mean age 70 Age range 51–78 >60 = 8 Inclusion = 4 2F/2M	The findings relating to the total knee replacement patients found that the surgery allowed resumption of valued, fun activities, and limitations were attributed to aging or other comorbid conditions.
Jeffery et al.^35^; UK	To understand patients' experiences of chronic pain following recovery from total knee replacement	Mixed methods including qualitative semistructured face to face interviews Thematic analysis	N = 28 (28 TKR) Mean age 76.45 Age range 57–87 Mean = 70 >60 = 22 14F/8M	The findings concentrate on the impact of pain after total knee replacement and how patients adapted their feelings about pain dependent on their individual context or situation. The authors suggested that poor communication from health care providers adds to patient distress and could be improved by surgeons adopting a more biopsychosocial approach
Johnson et al.^36^; UK	To explore pain relief use around the time of total joint replacement	Mixed methods with qualitative semistructured face to face interviews Phenomenological approach Inductive thematic analysis	N = 24 (TKR 10) Mean age 65 Age range 26–77 >60 = 17 Inclusion = 8 5F/3M	The findings concentrate on the patterns of pain medication use preoperatively, during hospital stay, and recovery at home after total knee replacement surgery. Pain medication use varies over time and is influenced by individual beliefs and advice from health professionals. They suggest that health professionals could play a larger role in optimising pain management.
Kleiner^40^; (THESIS) USA	To understand patient experience of pain after total knee replacement prior to hospital discharge	Hermeneutic phenomenology Face-to-face interviews on 1–2 d after surgery and day of discharge (3–4 d after surgery)	N = 15 (TKR 15) Mean age* Age range 66–86 >60 = 15 9F/6M	The findings highlight the progression over time of patients in the immediate postoperative period from a state of severe debilitating pain to reducing pain where greater function is possible. The author considers the payoff between enduring pain and obtaining function as suffering for a purpose.
Klem et al.^41^; Australia	To understand patient satisfaction after total knee arthroplasty and to identify what factors influenced their satisfaction	Mixed methods Constructivist grounded theory Face to face and phone interviews Coding framework	N = 40 (TKR 40) Mean age* Age range = 50–80+ >60 = 38 F/M #	The findings concern the meaning of satisfaction (ie, to gain improvement in symptoms or limitations) and categorization of these meanings. They show that patients can use various mechanisms to validate their individual experience and satisfaction levels. The authors suggest that greater satisfaction might be influenced by health care professionals to counter negative thoughts, feelings, and experiences.
Loth et al.^45^; Germany	To understand patient understanding of joint awareness by investigating bodily sensations and psychological factors raising patient's awareness of their knee	Mixed methods Phone interviews using a standard interview guideline	N = 40 (TKR 40) Mean age 69.1 Age range* >60 =† Inclusion = 40 F26/M14	The findings identify different situations that make patients more aware of their replaced knee. These include daily activities, specific movements, and the weather. There is also focus on bodily sensations and pain causing joint awareness and psychological factors that influence awareness. The authors suggest that there may be other ways to measure joint replacement success other than pain, stiffness, or functional scores.
Mahdi et al.^47^; Sweden	To capture patient experiences of discontentment after total knee replacement	Semistructured face-to-face interviews Qualitative content analysis with an inductive approach	N = 44 (TKR 44) Mean age* Age range 59–88 >60 =† Inclusion = 44 F/M #	Unfulfilled patient expectation leads to discontent or dissatisfaction. These are further broken down into unresolved issues and development of new problems eg, new pains, inability to function independently and the dissatisfaction with interactions between participants and health care providers. The authors suggest that health care professionals have a role to play in decreasing the gap between expectation and experience especially when communicating information regarding pain and function during recovery.
Maillette et al.^48^; Canada	To understand workers' experiences of work disability after total knee replacement	Narrative approach Mixed coding method with À priori codes Content analysis	N = 8 (TKR 8) Mean age 56 Age range 42–62 >60 = 2 0F/2M	The findings concentrate on disparity between expectations from surgery and the actual outcomes, fear of using the replaced knee, support needs for participants returning to work from health care providers and insurers and the reasons why they did or didn't manage to return to work. The authors suggest a need for more effective return to work rehabilitation practices and processes.
Marcinkowski et al.^49^; New Zealand	To describe the experience of adults with OA after total knee replacement	Grounded theory Unstructured face-to-face interviews 3 wk to 3 mo after surgery Constant comparison analysis	N = 9 (TKR 9) Mean age 71 Median age 69 Age range 54–85 >60 =‡ Inclusion = 9 5F/4M	The overall findings are summarised in a theme that considers participants thoughts of the future, returning to normality after total knee replacement. The subthemes describe enduring pain for some time, devising strategies for the process of recovery, and using inner resources to work through recovery. The authors suggest that outlining realistic recovery should be part of patient education for total knee replacement surgery.
Moore, & Gooberman-Hill^51^; UK	To understand why people don't utilise health care for chronic postsurgical pain after total knee replacement	Semi structured interviews Inductive thematic analysis	N = 34 (TKR 34) Mean age 74 Age range 55–93 >60 =‡ Inclusion = 34 18F/16M	The main finding with patients not seeking health care for chronic knee replacement pain is one of futility of action. This is further explained in terms of patients’ experiences with health care professionals, their expectations or risks of further treatment, treatment burden, acceptance of their situation, nature of pain, other comorbid conditions taking priority, and morals behind seeking further care. The authors suggest that health care professionals have a responsibility to help people access pain management and other appropriate treatment.
Pellegrini et al.^57^; USA	To identify barriers and facilitators to healthy eating and physical activity before or after total knee replacement	Semistructured interviews Constant comparative analysis	N = 20 (TKR 9) Mean age 61.7 Age range* >60 = 4 0F/4M	The main findings concern the facilitators and barriers to both healthy eating and physical activity. Specific barriers identified to physical activity included pain, functional limitation, and low motivation. Increased motivation and commitment to activity to increase function were seen as enablers. The authors suggested that improving mood and motivation could improve postknee replacement rehabilitation.
Perry et al.^60^; New Zealand	To explore patient perception of discharge home following lower limb joint replacement	Interpretive phenomenological analysis Interviews between 6 and 12 wk postdischarge	N = 11 (TKR 4) Mean age 76 Age range 66–88 >60 = 4 3F/1M	The findings concentrate on the lack of a shared decision on when to go home, the patients' dependence on family to go home and feel confident, the process of rehabilitation being trial and error, and interactions with health care professionals being paternalistic. The authors suggest that support networks are essential for discharge and more information would enhance the recovery process.
Sjoveian et al.^64^; Norway	To describe pain and rehabilitation in the first 6 wk after discharge from hospital after hip or knee replacement	Qualitative descriptive design. Semistructured interviews Qualitative content analysis	N = 12 (TKR 6) Mean age 68 Age range 45–83 >60 = 5 4F/1M	The findings are grouped under themes concerning pain on movement at rest, the need for support with activities of daily living and information needs on pain and exercise and follow-up on pain issues. The authors suggest that there is a need for more individualised support and information provision, especially by health care professionals for patients postdischarge.
Smith et al.^65^; UK	To explore patients' experiences and information needed for a decision aid for total knee replacement	Focus groups held pre- and postsurgery Framework data analysis	N = 31 (TKR 14) Mean age* Age range 50–89 >60 = 13 Inclusion = 13 F/M #	The findings concerning the postoperative period concentrate on whether expectation of surgery was met and feelings of abandonment after surgery. They also describe actual outcomes and cosmetic issues after surgery. The authors suggest information provision is key to helping future patients decide appropriately on surgery and that information on patient narratives would be one way to do this.
Specht et al.^66^; Denmark	To explore patient experience after fast-track total hip replacement and total knee replacement up to 12 wk after discharge	Phenomenological-hermeneutic approach Semistructured interviews and participant observation	N = 8 (TKR 4) Mean age 63 Age range 54–82 >60 = 1 0F/1M	The findings concern issues with the transition between hospital and home, pain and self-management of medication, issues around rehabilitation, including motivation and confidence. The authors suggest that greater individual involvement for patients in their discharge planning could influence pain management and recovery at home
Specht et al.^67^; Denmark	To explore patient experience after fast-track total hip and knee arthroplasty from the first visit at the outpatient clinic until discharge	Phenomenological-hermeneutic approach Semistructured interviews and participant observation	As above (same participant)	The findings largely concern patient experience of pain, their feelings of confidence or uncertainty around information provided, and their readiness for discharge home. The authors suggest that information provision is key to improving pain management before discharge home.
Stenquist et al.^69^; Dominican Republic	To investigate the impact of total knee replacement on physical activity for patients in a developing nation.	Semistructured face-to-face interviews content analysis	N = 18 (TKR 18) Mean age* Median age 66.5 Age range 34–80 >60 =† Inclusion = 18 F/M #	The findings concentrate on participants increased participation or resuming necessary and leisure/family activities, which were difficult prior to total knee replacement. Findings show participants have both concerns about using the joint and positive impacts of surgery on mental health. This study also notes a spiritual dimension to surgery. The authors suggest that it is important to note cultural setting and how this may impact on physical and mental health after surgery.
Webster et al.^74^; Canada	To explore reasons for engagement or lack of engagement in activities following total hip replacement or total knee replacement	Constructivist grounded theory Open-ended semistructured interviews Analysis by constant comparative approach	N = 29 (TKR 13) Mean age* Age range 38–79 >60 = 8 5F/3M	Findings for participants after joint replacement identify experiences of pain and mobility difficulties after surgery, comorbidities including mental health issues and painful joints, fears concerning the joint replacement, and the social context of recovery after surgery. The authors suggest that recovery is a multifaceted process and individualised approaches may enhance recovery.
Woolhead et al.^77^; UK	To investigate patients' experiences of outcome from total knee replacement	Interviews 3 mo presurgery and 6 mo postsurgery. Constant comparison data analysis	N = 10 (TKR 8) Mean age 64 Age range 40–81 >60 = 8 6F/2M	The findings highlight that almost all respondents reported continued pain and immobility and many struggled to make sense of this. There was self-blame for overdoing things after surgery. However, there were contradictory findings that coping abilities were better after knee replacement. The authors suggest that more sensitive outcome assessments are needed to make sense of individual patient experiences of total knee replacement surgery.
Wylde et al.^78^; UK	To understand assessment of persistent pain after total joint replacement	Face-to-face interviews Thematic analysis	N = 20 (TKR 10) Mean age 69 Age range 45–85 >60 = 7 3F/4M	The findings around the experience of total knee replacement identify the changing and fluctuating nature of pain and functional difficulty, comorbidity and other pains, and living with pain. The authors suggest that current generic pain measures are insufficient to capture the patients pain experience.
Zacharia et al.^82^; India	To understand Indian patients' expectations of and satisfaction of total knee replacement	Focus group discussion	N = 42 (TKR 42) Mean age = 63 Age range 60–65 >60 = 42 18F/24M	The findings consider patient satisfaction after surgery in respect of pain, range of movement, and independence. The study highlights a discrepancy between patient and surgeon expectation and the authors suggest that outcome assessments could better developed for these different populations.

* Unable to calculate mean age or provide range as individual participant details not provided.

† Data were selectively extracted for participants >60 but total number in sample >60 unknown.

‡ Range of ages given in study and SD calculated indicates >75% participants are >60 meaning all data were extracted.

>60, participants older than 60 y; F, female; F/M #, detail not given to identify split between females and males; M, male; N, number of total participants in study; TKR, total knee replacement participants.

### 3.2. Quality assessment

The CASP checklist^17^ scores were diverse ranging from 20^82^ to 27^41^ (Supplementary File 3, available at http://links.lww.com/PR9/A159). Study aims, method, and findings were generally well reported across the studies. Reporting of research design was variable; the research was identified as qualitative, but specific design was not consistently provided. This was where most disagreements occurred between the reviewers. One reviewer (C.M.) consistently rated this item as lower quality. However, discussion resulted in consensus on the lower quality rating. In most studies, the researcher's relationships with participants were rarely reported; this precludes evaluation of the presence/absence of potential undue influence. The reviewers had 76% agreement and PABAK of 0.52, representing moderate agreement.^19,42^

### 3.3. Qualitative meta-synthesis outcomes

Fifty-seven initial descriptive categories were identified in stage 2 of data analysis and were further refined at stage 3 with discussion amongst the authorship team producing 4 overarching analytic themes, with 13 descriptive subthemes. Synthesis of the included studies highlighted that the experience of pain and function following TKR were highly interrelated, thus themes and subthemes report data that integrate both concepts. The findings include all stages after TKR, ranging from immediately postsurgery to years afterwards. Table 3 highlights the included studies that contributed to each theme/subtheme with Table 4 providing key illustrative quotes.

**Table 3:** Study by theme and subtheme.

Study	Theme 1			Theme 2				Theme 3			Theme 4		
Subtheme	1	2	3	1	2	3	4	1	2	3	1	2	3
Berg et al.^5^; Sweden				X					X				
Bremner^9^ (THESIS) USA	X	X		X	X	X	X			X			
Bremner et al.^10^; USA	X			X		X	X			X			
Bunzli et al.^12^; Australia	X		X	X				X	X	X			X
Coutu et al.^16^; Canada					X		X						
Engström et al.^23^; Sweden	X	X	X	X	X				X				
Fletcher et al.^26^; UK	X				X			X	X	X			X
Harding et al.^32^; Australia		X		X				X					
Jeffery et al.^35^; UK	X			X				X	X	X		X	X
Johnson et al.^36^; UK	X						X			X			X
Kleiner^40^; (THESIS) USA		X		X	X	X	X		X	X	X		X
Klem et al.^41^; Australia	X			X				X	X	X		X	X
Loth et al.^45^; Germany				X	X	X		X					
Mahdi et al.^47^; Sweden	X		X	X	X	X	X	X	X	X	X		X
Maillette et al.^48^; Canada											X		
Marcinkowski et al.^49^; New Zealand	X	X	X			X		X	X	X	X	X	
Moore, & Gooberman-Hill^51^; UK	X			X	X		X	X	X	X			X
Pellegrini et al.^57^; USA		X											
Perry et al.^60^; New Zealand			X				X		X				
Sjoveian et al.^64^; Norway				X	X	X						X	
Smith et al.^65^; UK	X	X	X				X		X		X		X
Specht et al.^66^; Denmark	X		X				X					X	
Specht et al.^67^; Denmark			X				X						
Stenquist et al.^69^; Dominican Republic	X				X				X	X	X		
Webster et al.^74^; Canada	X	X	X					X	X				
Woolhead et al.^77^; UK	X		X	X	X		X	X	X	X			
Wylde et al.^78^; UK	X				X		X	X		X		X	
Zacharia et al.^82^; India	X								X				X
Number of papers included in sub theme	18	8	10	14	12	7	13	12	16	14	6	6	10
% of studies included in sub theme	64.3	28.6	35.7	50	42.9	25	46.4	42.9	57.1	50	21.4	21.4	35.7

Study is featured in a theme/subtheme if marked with “X.”

**Table 4:** Illustrative quotes.

Themes and sub themes	Quote
THEME 1: “Magic, it got better”^65^: Recovery from TKR	
“Yeah, very worthwhile”^77^: Positivity with less pain and returning to activity	“[I'm satisfied] because I've got more movement and less pain … I can do all the activities without as much pain as I used to have.”^41^ “I came here without much hope. I could not believe that I got pain relief.”^82^
“When everything turned”^23^: Defining and noticing recovery	“It took a long time to get better [total]. I went back for my 6-mo check-up, and about a month before I thought “I really wish I hadn't had this done,” it was so painful. And literally a fortnight before I went to see him [surgeon] suddenly, magic, it got better.”^65^ “…I got up today and walked all the way up to the nurse's station and back and it wasn't too bad…. I guess that's ‘cause I hadn't been, you know, walking that other was the first time I walked”^40^
“Everybody has a different sort of frame”^12^: Recovery norms	“I think it was not knowing what I should feel or what stage it should be progressing at. I understand that everybody has a different sort of frame of what happens and how it takes place, but I just felt that I was not told enough as to what to expect from it. I thought a matter of six weeks and I'd be running around like a champion again. But basically, it has been nearly 12 mo and I really feel that I'm only getting the relief and benefit from it now.”^12^ “You sort of get this book and it tells you what exercises to do, and I done all them and it says after 3 wk you must come off your sticks and you can bear weight and after 6 wk you should be able to walk up and down the stairs normally … well I can't walk up and down the stairs normally after 6 mo”^77^
THEME 2: “Amazing pain”^40^: The pain experience, beliefs, and impact on function	
“A real bear”^40^: Severe pain that impacts function	“The pain is unbelievable. If I don't hang onto things, I'll fall … It's almost to the stage where I scream because it's so painful and [when] I finally get up and then, you know, sort of walking – It's only very slow and I've got my walker with me and it's a high one that I lean right over … I try to take one step at a time, and I've got to be very, very careful because I will fall over if I'm not careful, so you know, very difficult getting around.”.^41^ “Oh, I kinda looked for it; people was telling me that it would be a real bear and it was.”^40^
“Good days and bad days, good nights and bad nights”^78^: Variation in pain and impact on function	“It's really that you get good days and bad days, good nights and bad nights” (P5) …You have good days and bad days on that one” (P9)”^78^ “I don't like the sharp ones, when I get them that is awful, you know, but I do tolerate it with medication, because, like I said it's erm, it's a breeze to what it was, yeah.^51^
“Aches and aches and aches”^10^: Discomforts	“At night [my knee] just aches and aches and aches and aches.”^10^ “Oh yes, I suppose for about 3 mo I was tired. You haven't the energy, the energy isn't there and you try and you get so tired. I hadn't any idea about the tiredness.”^49^
“Trial and error”^60^: Individuals managing pain and function within contextual beliefs	“So you can't divorce pain from individual people's mindsets. You can't. And in my case, I say probably it might have something to do with my age, my upbringing, this kind of thing. Nobody in my family was pill-ish… I may be wanting to endure a bit more pain and make it seem small to you rather than be seen to be dependent upon [Percocet]. Dare I say I'm proud that I'm not dependent on that? I'm telling you with pride that I'm not dependent on this.”^9^ “Well I don't like taking them. … and I just felt in so much pain I just had to take it. I wouldn't have walked otherwise; I wouldn't have got out of the bed.”^36^
THEME 3: “I just live with it”^35^: Struggles after TKR	
“You think it's gonna be so much better”^45^: Mismatch of expectations and outcomes	“Yeah, it's been a year. It's just that …I've had this goal the whole time. I've complained a bit, and then he'd [doctor] say that it’s only been this and that. Yeah, yeah, Okay. But now it's been a year., but it is annoying. Because it hurts and I feel,…mm, the longer time I used it the more pain it cause!”^45^ “…It's disappointing because you think it's gonna be so much better after you've had it done, and really you're not, it's different but you're not the same as you were before, you've not got the problems that you had before but they've been replaced by other problems…it really has made me more handicapped than I was before”.^35^
“You're not getting anywhere”^74^: low mood, depression, anger, and fear	“You just get a little depressed about it at times, I guess, it just feels like I'm not going to get there. You kind of think, okay, is it going to be like this for the rest of my life or what, or can something be done… it is depressing … you're not getting anywhere, that's the thing. There's stuff you want to do and you can't do it.”^74^ “One is afraid to do things with the knee that was not operated on, and that it will affect the knee which was operated on ….”^23^
“A balancing act”^9^: conflicts, choices, and trade offs	“It was just like this awful balancing act, how much pain can I stand before I have to ask for more pills “^9^ ”…it's improved from how I was greatly in everyday just walking around and you know just doing general stuff but as far as the things that I really love to do I still can't really do them – or I can do them but to a lesser degree.”^41^
THEME 4: “I don't want sympathy”^35^: Individual interaction with others	
“You look different altogether”^49^: Impact on social functioning	“This lady said to me the other day when I went round to her house to do a job, ‘Gee you look good. Your face isn't drawn with the pain. God, you look different altogether. So I'm rapt.”.^49^ “I used to be comfortably over 6 feet, but I'm not any more…your legs go like a jockey's…you walk around in your best suit…people say, ‘What's happened to your leg sort of sticking out at a funny angle?’”^65^
“I have to rely on other people”^78^: Support needs	“I don't want sympathy I just want um, practical help if I need practical help, because all the sympathy in the world is not gonna make it go away or make any difference.”^35^ “I don't do family activities. The only social I do is go up these small clubs for bingo but I can only go up them if somebody comes to drive me up and drive me back so I have to rely on other people to do it for me. So in a round about way I will just say no to that because I have to rely on other drivers”.^78^
“Once they're done that's it,”^35^: Suboptimal interactions with health care providers	“. . .you don't feel as if uh, not backing you but as if they're um not interested anymore, once they're done that's it. “.^35^ “It should be so that the hospital and the doctor call me and ask how I am doing, it would be easier for me as a patient to avoid sitting on hold a whole morning. It would in any case be desirable the first time afterwards and it would give a more personal contact, which would alleviate my concerns.”.^23^

#### 3.3.1. THEME 1: “magic, it got better”: recovery from total knee replacement

This theme^65^ covers recovery from the early stages post-TKR surgery to later stages, including resumption of activity. It encompasses 3 subthemes that describe patient's (1) positive surgical outcomes of pain and function, including noticing mood changes, (2) defining and noticing recovery, and (3) describing how experience contrasts with understanding/expectation of recovery norms.

##### 3.3.1.1. Subtheme 1: “yeah, very worthwhile”: positivity with less pain and returning to activity

Although several studies found adverse outcomes or reasons for discontent with TKR,^12,35,41,47,51,78^ others found positive outcomes, with quick recovery,^65^ gaining pain relief and restoration of function leading to improved quality of life.^23,41,77^ Total knee replacement was considered “very worthwhile”^77^ especially when framed against discontent prior to surgery.^69^ Happiness^82^ was expressed in the weeks and months after TKR^9,10,36,65,82^ due to restored functional capabilities (eg, participating in gardening)^74^ and minimal or no pain^9^ with some “eventually pain free.”^65^ Even reduction of pain, without complete resolution, led to improvement in the quality of life^23^ after TKR.

Whilst some remained hampered by other painful joints, they were able to “get about with not having the pain”^65^ of their previously osteoarthritic knee. Even if range of movement did not improve, pain relief alone brought improvement in daily activities,^82^ creating reduced “barriers to activities”^26^ from “more movement and less pain.”^41^ They particularly valued returning to sport,^66^ reconnecting with social^41^ and functional daily activities^69^ of family life,^41^ going back to work,^23^ and resuming use of public transport.^49^

##### 3.3.1.2. Subtheme 2: “when everything turned”: defining and noticing recovery

After TKR, some participants struggled to understand what recovery really meant, and how they might notice it.^23^ They attempted to define it; “recovery to do what? … have a shower … drive cars … walk a mile … ride a bike 5 miles …”^65^ Motivation to work towards recovery was not universal, and despite opportunities for rehabilitation,^57^ some participants found it difficult to motivate themselves.^74^ However, signs of recovery such as ease of daily activities encouraged some participants to continue to challenge themselves during recovery^23^ with some considering challenging activities, like managing stairs, as functional training.^23^ As healing continued after TKR, increased levels of activity became easier even when pushed (eg, physiotherapy),^40^ pain lessened, and medication consumption reduced.^9^ Some participants noticed a specific time point when they were aware that recovery was progressing. This occurred for some in the relatively early postoperative days.

“…the first day you thought you were in hell, the second day you knew you were and then after that it got better. And that's true.”^40^

Others took longer, with months passing until the magical^65^ time came “when everything turned and it started to feel better and better,”^23^ taking their new joints “for granted.”^49^ They no longer needed to plan movements, which was a marker of improvement, “I don't think I just do it.”^32^ Examples of activities that were resumed following TKR are provided in Table 5.

**Table 5:** Functional activities resumed and not resumed after total knee replacement.

Functional domain	Activities resumed after TKR (when previously difficult)	Activities not resumed or remained problematic after TKR
Activities of daily living	Toileting, using low level Indian (squat) toilets, dressing, cooking, and getting out of bed^69,82^	Using a squat toilet,^82^ getting down on the floor,^65^ picking things up from the floor, exiting the bath^26^
Mobility	Walking, stairs^32,69,82^	Going up stairs,^74^ rising from chairs^47^ kneeling,^26,45,47,65,77^ walking—painful,^41^ slower,^32,49^ lack of endurance,^47,69^ walking with crutches—lack of endurance^47^ and inability to carry items^69^
Social	Social activities, family time, community participation, spiritual (church) and hobby activities,^32,41,69,82^ independent excursions, driving,^49^ holidays, going out, and travelling^23,32^	General social interactions and activities^49^ Leisure and sports, including, dog walking,^41^ camping,^26^ gardening,^26^ horse riding,^65^ dancing,^47^ hunting,^47^ fishing,^47^ playing golf,^47^ skiing,^47^ hiking,^47^ swimming, picking berries in the forest,^47^ cycling^47,74^ Returning to work,^48^ community/voluntary activities,^26^ family time and engaging with grandchildren,^26,47^ going shopping,^77^ spiritual activities like “praying in church”^26^

TKR, total knee replacement.

##### 3.3.1.3. Subtheme 3: “everybody has a different sort of frame”: recovery norms

Whilst there was a general expectation of recovery, including resolution of pain and return to function, what was normal was unclear to participants.^12^ Recovery started in hospital, with some feeling great^66^ and ready to go home almost immediately after TKR,^67^ although others were not medically ready.^47^ When progress was made according to individual's expectations after surgery,^23^ they were satisfied; however, some were not content. Although some health care professionals had suggested recovery times to participants of around 12 months,^12^ others described their annoyance regarding the lack of information on recovery norms, specifically timeframes after TKR.^12^

“everybody has a different sort of frame of what happens … I was not told enough … I thought a matter of six weeks and I'd be running around … it has been nearly 12 months … I'm only getting the relief and benefit from it now.”^12^

Some accepted slow recovery,^77^ but when there were misconceptions around resolving pain and regaining function,^12,47,49,74^ there was frustration and impatience^23,77^ associated with participants' functional difficulties.^65^ Misplaced expectations of recovery norms meant that some participants forced activities in their recovery.^77^ Some participants pushed hard to gain function and meet their expectation of where they thought they should be and then worried they had damaged the TKR by doing too much.^77^ Others had confidence in their own abilities to recover^49^ without health professional involvement that led to devising personalized home-based rehabilitation programs and feeling in charge of their diminishing pain and improving functional outcomes.^60^

#### 3.3.2. THEME 2: “amazing pain”: the pain experience, beliefs, and impact on function

This theme describes the patient pain experience from the early days after surgery to coping at home after surgery.^40^ Four subthemes emerged that describe the severe pain that impacts function, the variation in pain and its impact on function, discomforts that contribute to the pain experience, and the use of medication in context of beliefs and attitudes towards medication itself, as well as towards pain and function.

##### 3.3.2.1. Subtheme 1: “a real bear”: severe pain that impacts function

Total knee replacement was typically a painful experience^5,9,10,40,64,77^ with most experiencing severe pain in the first 1 to 2 days after surgery,^40^ easing over a period from 3 days to a few weeks.^23^ Some experienced relapses in pain^12^ but dealing with pain generally became easier over time.^51^ Few were without surgical pain.^9^ Some were prepared for pain by their surgeon,^35^ but others reported more pain than expected.^9^ For those who were unprepared, intensity and duration of pain was alarming, creating “significant psychological impact.”^12^ They experienced distressing negative emotions, with night and resting pain “uncomfortable and worrisome,”^47^ and some were so distressed that they wanted to “scream because it's so painful.”^41^ High pain levels surprised participants who self-reported as having high pain tolerance.^9^

The severity of immediate postsurgical pain was described as “a real bear,”^40^ “horrible,”^23^ “amazing … unbearable,”^40^ and so bad it impacted breathing and talking.^40^ They rated pain as extreme,^40^ “On a scale of 1 to 10, [the pain] was about 15.”^9^ Participants described TKR pain as the worst amongst all previous surgeries.^9,40,77^ However, some found it difficult to communicate the nature and level of their pain and suggested that only other TKR patients could truly understand their experience.^40^

Acute postsurgical pain that limited movement^40,64^ worried many participants, making them want to stay in hospital.^40^ Participants described “crackling,”^45^ “cutting and burning,”^23^ “sharp, shooting pain that burns,”^40^ “stinging pains and burning sensation,”^64^ “nagging and aching pain … as a barbed wire inside the joint,”^64^ and getting out of bed made some people feel like their leg would break.^40^ Participants noted prolonged increased pain after TKR^47^ or pain that occurred in the daytime, when before surgery they only had night pain. Some people became “more handicapped” than before TKR,^35^ noting more effect on their daily activities than before. They doubted that they would function normally and struggled with rehabilitation in and out of hospital.^41^

The longer they were affected by severe pain, the more frustrated and worried people became.^47^ Participants with “long-lasting pain, swelling, stiffness, and clicking sounds,” or loss of sensation^47^ were anxious. They worried about poor surgical technique, insertion of an incorrect “metal bone,”^41^ poorly positioned or loose prostheses, inflammatory or cancer-causing materials, and the potential for fractures, infection, and thrombosis.^47^

##### 3.3.2.2. Subtheme 2: “good days and bad days, good nights and bad nights”: variation in pain and impact on function

Pain varied in severity, intensity, nature, and duration amongst participants^9,23^ from day to day and night to night.^78^ Participants hoped that they would have a steady decline in pain, where the severe pain would reduce to a “… normal amount of pain and normal amount of discomfort,”^40^ but this was not always the case. When thinking about pain, participants considered the nature of pre- and postoperative pain to be of different variants.^23^

Changes in activity meant pain levels also varied at rest and with movement,^64^ increasing and decreasing depending on what they were doing.^16,45,77^ This variability impacted people from early postoperative days;^40^ sometimes affecting daily function (ie, walking^47,51,78^) long term,^69^ which was disappointing and annoying.^45^ Some participants developed avoidant strategies when anticipating increased movement-related pain and so laid motionless in bed.^40^ Pain was also experienced in static positions, such as standing,^64^ standing after resting or sitting,^45^ and when kneeling.^26^

##### 3.3.2.3. Subtheme 3: “aches and aches and aches”: discomforts

Participants noted 5 main areas of discomfort during their recovery after TKR: discomfort leading to problems sleeping; discomfort in bed; tiredness and fatigue; stiffness; and unpleasant sensations.^10^ Sleep problems^9,40,64^ included nocturnal aches^10^ and disruption from sequential compression devices used in hospital. Compression devices and cold packs on the knees forced participants to lie in fixed supine positions. The inability to reposition in bed gave participants problems managing bedding, maintaining a comfortable temperature, and they experienced back and buttock pain.^40^ One participant reported exhaustion persisting for months after surgery.^49^ Complementary and prescription medication aided sleep.^9^

Reduced mobility overnight brought morning stiffness^40,45,47^ with swelling in both the knee joint and muscles.^64^ Stiffness was felt both soon after TKR and as a chronic problem.^47^ Medication^40^ and being able to “walk around”^45^ combated pain, swelling, and associated stiffness.

Unpleasant sensations remained; “sore … some numbness”^64^ or “aches, … soreness … or discomfort.”^9^ Participants also described weakness^45^ and heavy sensations in the operated leg.^40^ Some unusual sensations “encompassed the whole knee,” it felt “strange,”^45^ and “did not feel the same as before.”^47^ Sensations were also affected by the weather, with the operated knee feeling colder in the winter.^45^ Even when immobile, a few participants could detect unpleasant sensations^40^ and some perceived increased sensory awareness of the operated knee during a variety of functional movements.^45^

##### 3.3.2.4. Subtheme 4: “trial and error”: managing pain and function within contextual beliefs

Return to function was inconsistent, individual and “trial and error.”^60^ Participants adapted movements^16,65^ because of persistent pain and reduced mobility, creating workarounds.^77^ Medication use was also trialled by individuals, outside of clinician recommendation, such as using complementary medications, reducing doses, or stopping medication.^10,36^ Effective pain relief enabled some people to cope with TKR pain,^36,67,78^ thus aiding functional movement, physiotherapy, and sleep.^9,10,40^ However, some participants feared reliance on medications^9^ and therefore underplayed their pain and distress to health care professionals.^36,40^ Analgesia was often consumed before activity, but not always afterwards when pain returned, demonstrating stoicism^36^ or acceptance of postactivity pain. Participants also persevered without analgesia^10^ pushing through painful activities (therapy).^40^ Declining medication was explained in context of their social situation,^35^ pain levels, pain management beliefs, avoidance of opioids,^10^ mindset, upbringing, or to allow natural healing to occur.^9,36,40^ However, when participants took analgesia, despite a preference not to,^9,51,66^ they reported feeling overwhelmed by pain^36^ or under personal^66^ or health care provider pressure^40^ to accept medication. Participants perceived pressure from health care professionals to take medication immediately after TKR^40^ and for chronic post-TKR pain.^51^

Participants were disappointed when pain relief was not timely^40^ or effective^47^ either when provided in hospital^40^ or with pharmacy purchased (over-the-counter) medication.^9^ Participants sometimes perceived that health care staff withheld analgesia to assess their progress^40^ by seeing if they could function without it. Complementary medicines “really helped” ^10^ some participants in conjunction with other nonpharmacological methods for pain management, including “ice, warm compresses, exercises, leg elevation, self-massage, and distraction.”^9^

#### 3.3.3. THEME 3: “i just live with it”: struggles after total knee replacement

This theme expresses the negative outcomes after TKR. It encompasses the difficulties experienced after surgery, low mood, and negative emotions occurring due to pain and functional issues; balancing the problematic aspects of TKR with positive outcomes; and enduring ongoing pain and functional limitation.^35^

##### 3.3.3.1. Subtheme 1: “you think it's going to be so much better”: mismatch of expectations and outcomes

Participants' general expectations were that most difficulties after TKR surgery would resolve over time,^26^ but some found things “did not improve during the first year as expected.”^45,47^ Expectation was that TKR would result in having a “normal” knee^26^ and be “so much better” after surgery.^35^ Some functional limitations, for example, kneeling, were experienced immediately after TKR and persisted for at least a year or more.^12,26,35,45^ Some participants reported insufficient bend in the operated knee^26^ or increased swelling and pain^45^ with more vigorous activity, consequently limiting function. Participants also described pains occurring in other body parts (back or hip or foot or other knee) after TKR,^47^ although whether these were a direct consequence of the surgery was unclear. Pain and functional restriction^32,41,45,47^ meant that participants could not participate easily in things that they had anticipated enjoying after TKR, such as sport, community activities, and work (Table 5). However, numerous studies^12,26,32,41,45,49,51,74,77,78^ indicated that diminished function and reduced activity after surgery were not always due to pain but instead due to priorities, misunderstanding advice, comorbidities, and low expectations (Table 6).

**Table 6:** Reasons provided by participants for low function and inactivity after total knee replacement.

Age^12,32^
Other painful body regions or joints: back,^41^ hip,^45^ other knee^51^
Other comorbidities; poor lung function^78^ being overweight^32^
Were advised not to attempt certain activities (kneeling)^26^
Were “content”^32^ with reduced activity levels as they were able to participate in important social and family activities^69^
Low function and accumulated losses prior to surgery^26,47,57^
No expectation of potential capability as it was not discussed in presurgical consultations^26^
Expecting generalised rather than specific improvement in pain and mobility^35,51^

##### 3.3.3.2. Subtheme 2: “you're not getting anywhere”: low mood, depression, anger, and fear

Low mood and negative emotions (anger, annoyance, anxiety, frustration, fear, depression, hopelessness, disappointment, regret, discontent) were consistent findings^5,12,23,26,35,40,41,47,49,69,74,77,82^ both immediately after TKR and in the longer term. Shortly after surgery, where there was uncertainty around resolution of surgical pain, some participants felt close to “a sort of a breakdown.”^12^ Anticipating sudden “shooting pain”^49^ on movement was frightening. Some described hopelessness because of poor post-TKR pain management.^5^ Even when participants reported they were pain free, some remained discontented due to the “unpleasant pain experience in the first months” after TKR.^47^

Some participants regretted undergoing TKR^23,40^ particularly when they noticed “deterioration or no change in their situation”^35^ and similar pain to presurgery levels. They compared their outcomes adversely to others^41^ and felt “… depressed … you're not getting anywhere …. There's stuff you want to do and you can't do it.”^74^ Negative thoughts of the future and the potential for persistent problems led one participant to suicidal ideation.^35^ Several participants worried about future scenarios of falling^26,41,49,69,74,77^ not being able to get up^26,69^ and what the future implications of this might be.

Fear of using the operated knee^12,23,26,47,60,65,69,74,82^ made participants “extremely cautious”^60^ and some hoped by restricting use that the TKR would “last” longer.^12^ When participants felt pain from overexertion, they berated themselves.^23,60,77^

“… have I been stupid and done something silly, I didn't know whether I had done some damage … cos I did go mad when I came home … really I should have rested it.”^77^

Therefore, to prevent damaging the prosthesis they restricted everyday activities, sports, and hobbies.^12,23,26,65,69,74^ However, some were anxious over even simple functional movements that meant they did not follow exercise advice, leading to persistent functional restriction, “I was told to flex my knee on day 3. But I hesitated … now I am unable to flex the knee more than 50°.”^82^

Several participants hoped for additional support to help with pain, functional and emotional issues including physiotherapy,^51^ or someone to talk to.^23^ However, some did not seek further help as they thought it was futile^26^ because they perceived: there were no options beyond further medication or surgery;^51^ they were “bothering” health care providers;^51^ surgeons had others worse off than themselves;^51^ further surgery could worsen their situation increasing pain or further reducing mobility;^47^ and some options for resolving outstanding issues were expiring due to advancing age.^51^

##### 3.3.3.3. Subtheme 3: “a balancing act”: conflicts, choices, and trade offs

Participants expected to trade off the short-term surgical pain after TKR against their long-term goals of improved function and reduced OA pain.^40^ Continued limitation by pain or reduced function after TKR^12,35,41,47,69^ or limited function despite improvements in pain and stiffness^12^ meant that people had to balance conflicting needs by making specific trade-offs and accepting negatives with the positives^9^ (Table 7). Numerous side effects of medication^9,10,36,40^ were balanced against experiencing pain and consequential functional restriction.^36^ For those with chronic pain, they had “learnt to live with it,”^78^ with “stoicism, framing their situation in a positive light”^35^ and explained that they needed to “get on with it,”^49,51^ accepting it^35^ and continuing to undertake activities even with difficulty^78^ rather than choosing further intervention. Greater acceptance of limitation and reduced distress occurred over time.^26^ Some people reasoned outcomes by comparing with others who they saw as “worse off”^41^ meaning that they accepted imperfect but comparatively good outcomes^26,41,51,77^ with others. However, for some people, such comparisons made them unhappy with their outcomes.^77^

**Table 7:** Examples of trade offs and compensations.

Desired function (not achieved)	Actual result: trade-off or compensation
Kneeling	Pain relief^26^
Pain relief	Did not need to use a wheelchair^26^
Kneeling	Bending at waist/using pick up stick^26^
Bathing (using a bath)	Showering^26^
Gardening at ground level	Installing raised beds^23^
Normal mobility without aids (crutches)	Scooting on a chair with wheels (not a wheelchair), using non-slip footwear^64^
Independent living	Asking family members and using paid assistance^26,49^

#### 3.3.4. Theme 4: “I don't want sympathy”: individual interaction with others

This theme describes the interactions of TKR patients with others in social, family, and health care settings. It has 3 subthemes: impact on social functioning and appearance to others; the need for supportive care; and the quality of health care provider interactions.^35^

##### 3.3.4.1. Subtheme 1: “you look different altogether”: impact on social functioning

People were concerned about social functioning and their appearance after TKR. Some felt happy to appear “different altogether”^49^ because they felt better, due to the lack of pain. However, others did not want to be seen by others as being in “misery”^40^ after surgery. Cosmetic appearance was also important causing upset when their appearance was remarked on, such as loss of height, visible scarring, deformed appearance (“sticking out at a funny angle”),^65^ or having a larger knee, especially when the surgeon promised “a better-looking knee.”^47^ Participants worried about how they appeared at work, particularly if they would need to functionally rely on colleagues, who might “complain.”^48^ They were fearful of inability to return to current work or being unable to find a new career^47^ due to TKR.^48^ Others were prepared to challenge perceptions of their function and demonstrate their fitness to resume work.^69^

##### 3.3.4.2. Subtheme 2: “I have to rely on other people”: support requirements

People needed extra support after TKR.^79^ Preference was expressed for individualised support after TKR “that considers their condition in the context of their lives.”^35,78^ Practical support was valued over sympathy for pain or functional limitation.^35^ Additional help was needed for ADLs, such as “personal hygiene, dressing, or kitchen work,”^64^ especially whilst on crutches. Support was typically provided by family members^64^ and paid help, such as cleaners.^78^ Family support was preferred by some over community services,^64^ with participants finding their “grandson and … my doggy,”^41^ and their “daughter”^66^ to be motivational. Social networks provided support during convalescence^49^ with emotional support provided for pain helping participants accept their experience, “… most of our friends have got pain anyway.”^35^ Those with limited support scaled back family and social activities so they needed less help.^78^ This need to accept help challenged participants' desires to be independent as they had to “ask people or accept when people offered.”^49^ Participants specifically relied on others for transport, which was problematic depending on the vehicle. Accessing some cars was functionally difficult and painful, “… some cars I can get out of quite easy and other cars I have to twist my guts to get out and that twists the knee and then I am 10 times worse.”^78^

##### 3.3.4.3. Subtheme 3: “once they're done that's it”: suboptimal interactions with health care providers

Positive findings and descriptions of empathy from health professionals towards participants were rare, with several descriptions of suboptimal encounters.^35^ Having said this, there was some valued pain management support^36^ that encouraged some participants to persist with painful therapy.^40^ During hospital stays, some participants felt unsupported by nursing staff when medications were not administered when required, when medication was administered forcefully, or “nurses forced them to move even when they were in severe pain.”^40^

Aftercare by doctors and physiotherapists was criticized,^35,47,51,65,82^ and some participants lacked trust in these professionals.^47^ Several blamed delayed physiotherapy,^47^ unmotivational physiotherapists,^35,47^ and junior doctors^82^ for suboptimal outcomes. Support after TKR was rarely offered,^35^ and when participants sought additional intervention, the main offerings available were surgery^35^ or more medication.^51^ Without additional information to support recovery, participants experienced a “sense of abandonment.”^65^ Even when some information was given (exercise sheet) and further promises of support made, they rarely materialised;

“After the operation I asked about physiotherapy, and they just gave me a sheet of paper and said do these exercises. When I queried this, they said, um, that I'd have to take up physiotherapy with the consultant, at my next appointment. They never made me an appointment, I've never seen the consultant from that day to this.”^35^

What they wanted was proactive post-TKR contact,^23^ information, practical advice, and psychological support.^35^

Participants felt unheard by health care providers who were uninterested in their problems^26^ after TKR; “once they're done that's it.”^35^ Surgeons' views were often discordant with participants when assessing the outcome; some surgeons did not acknowledge participants pain and functional problems, “… he said well there's nothing wrong and I said well tell my knee that please.”^35^ People felt disgruntled when surgeons told them “Everything's quite normal.”^51^ When recurrent knee pain was explained by health professionals as referred pain from other body parts^12^ participants remained disappointed and unconvinced. Participants felt that their surgeons were preoccupied with bending of the knee joint, appearance of the joint on X-ray,^35^ and the cosmetic appearance of the scar^41^ as opposed to acknowledging persistent pain.^51^ There was no recourse to anyone else^35^ and the lack of investigation, explanation, or follow up^12^ from surgeons frustrated participants;

“I wanted some, you know for him to say it could be this, could be that, but no. Well it should be all right, I've [the surgeon] done everything properly, and that was it.”^35^

## 4. Discussion

This meta-synthesis summarised patient perspectives of pain and function after TKR, identifying 4 key themes with important prognostic and management implications. We found that pain and function were interdependent but not synonymous. While functional limitations were typically present when pain persisted, function was not solely underpinned by pain. Overall, we found that presurgical information provided about TKR and postsurgical support provided after TKR were inadequate for many people, with timely individualised support lacking when things do not go as expected, resulting in life-affecting consequences.

The need for more information about the expected outcomes and timeframes after TKR was identified across most themes. Theme 1 highlighted gaps and inconsistencies in patients' understanding of recovery, including timeframes for normal resolution of pain and resumption of function, which ultimately influenced their perception of the operation's success. Theme 1 also showed that people undergoing TKR do not always receive information about potential negative surgical outcomes of chronic pain or long-lasting restricted function. While current TKR presurgical management typically includes education, our findings suggest inadequacy in the quality of educational information being provided. There is evidence that improved surgical outcomes occur when preoperative patient education about TKR is undertaken,^21^ ideally via both consultation with health professionals and educational materials, with the latter made available in numerous formats (written, videos, etc.).^38^ Relying upon potentially misleading online information,^63^ accessing “Dr Google,”^38^ or recalling preoperative consults^27^ is not ideal. Patients require nonbiased clear information on the ranges of “normal” recovery on which to realistically base their expectations.^27^

Theme 2 identified large variability in experiences of pain and function after TKR with some reporting immediate benefits and others reporting delayed or absent benefits or continued fluctuations over time. Severe postoperative pain was unexpected, different in nature to presurgical pain, and sometimes mismanaged by health professionals, resulting in anticipatory fear of moving. Understanding and communicating this “normal” variability to people undergoing TKR is key to addressing fear, distress, and other negative emotions that often occur when unprepared and blindsided by unexpected experiences (themes 1–3). Documents/infographics that illustrate the varied trajectories of “normal” recovery (and when to seek help) seem a relevant priority for provision preoperatively. Understanding variabilities in recovery may identify important subsets of people for whom different management is necessary for optimal outcomes. Theme 2 highlighted that some people still experience significant movement-induced pain following TKR. Impaired pain system function (eg, enhanced facilitatory processes and inefficient inhibitory processes) can occur in knee OA and influences movement-evoked pain.^22^ These impaired processes may be relevant targets for differential treatment (eg, medications with enhanced efficacy in those with intact inhibitory processes;^80^ exercise to enhance inhibitory processes^14^). Theme 3 identified additional types of sensations or discomforts that people experience following TKR that may not be expected or considered in current management, such as poor sleep contributing to discomfort post-TKR. Given important links between sleep quality and surgical recovery,^28,46^ improved sleep as a purposeful clinical target may be warranted. Finally, given differences in the *nature* of pain pre-TKR vs post-TKR identified here (theme 2), future research should include an assessment of *both* pain intensity/severity and nature, as focus only on the former may miss critical data relevant to prognosis, such as the presence of neuropathic-like pain components.^61^

This meta-synthesis identified that a subset of people are highly fearful after TKR and avoid activity. Activity avoidance appears based upon both the pain experience and inaccurate or unhelpful beliefs, eg, activity is going to damage or wear out the prosthesis. Unhelpful beliefs about OA knee vulnerability reduces engagement in activity;^11^ our findings suggest that this effect extends to the prosthesis and the postsurgical period. Importantly, targeting such unhelpful or inaccurate beliefs about knee OA with pain education shows preliminary benefits for pain, function, and activity levels.^68^ Considerable gaps in the provision of pain management information have been identified, the resolution of which would likely enhance the patient recovery process.^38^

A key finding of this review is that valuable access to individualised services following TKR is currently inadequate and largely absent. The struggles and compromise after TKR (theme 3), which include disappointment, regret, depression, anger, and anxiety, support the need for improved TKR clinical management. For instance, improved guidance for patients about when pain and other symptoms (noises or sensations) are a normal part of recovery or when they are a cause for concern, including signs or symptoms associated with prosthetic damage. Patients also need information about the implications of forcing the pace of recovery by “overchallenging” function (leading to pain flares), concurrent with understanding the likelihood of damage to the TKR from such actions and what to look for to independently track their progress. Improved prognostic and safety information could have 3 outcomes: empowering patients to know what to expect and look out for; increasing patient satisfaction by showing their outcomes fit into the range of “normal” recovery; and, finally, alerting clinicians to the need for further intervention when patients fall outside of recovery norms. Importantly, theme 3 highlighted that people who need help the most may not ask for it. Thus, in-built systems are needed to identify when suboptimal outcomes occur (pain, function, and/or mental health), normalising the inclusion of intensive rehabilitation, group rehabilitation or support programs, or psychological referral after TKR. The traditional practice of operating on the knee, having a few short postsurgical follow-ups, and letting people “get on with it” does not appear sufficient when considering patient perspectives.

A critical aspect identified was patients' profound discontentment with communication and relationships with their treating clinicians. Identified in theme 4, people undergoing TKR reported that they did not feel heard, with their concerns about progress downplayed or ignored, without referral to other relevant management options. Patient experience within the health system is a known contributor to clinical outcomes,^20,27^ with poor communication often underpinning patient-led complaints, negative feelings, inability to comply with treatment, and stressing the health care professional and patient relationship.^43^ Deficient patient and clinician relationships may also exacerbate mental health issues identified (themes 1 and 3), accentuating depression and anxiety, and possibly influencing pain catastrophising, all of which are known to have associations with pain severity^4,7^ and suboptimal orthopaedic functional outcomes.^30^ There has been minimal investigation of mental health issues in this population. With evidence for preexisting mental health issues and poor outcome following TKR,^56,59^ and poor mental health remaining despite relative improvement in pain and function,^58^ this suggests that a more collaborative and supportive approach between clinicians and their patients could also boost mental health and impact postoperative outcomes. Providing individualised support following TKR may help target low mood and anxiety: it may assist patients in identifying improvements that, as theme 1 highlighted, may be missed if not pointed out (eg, realising that you can now do more with the same level of pain). In addition, mental health supports such as cognitive behavioural therapy (CBT) reduces kinesiophobia and pain after TKR^13^ and psychological care (education/reassurance) provided throughout TKR improves negative mood, promoted hope, and resulted in superior clinical outcomes.^81^

This meta-synthesis highlights the need for formal collaborative exploration of required practical support in context of individual situations and independence. Improved patient-clinician relationships and trust may also be key to issues raised in theme 2 surrounding use of analgesics, including combating stigma and beliefs related to perceived overreliance on medication. Planning, and importantly, communicating the plan for medication tapering may help people feel reassured that they are not becoming reliant on medications but rather that use is appropriate. Preliminary work has shown that preoperative medication use (serotonin selective uptake inhibitors) for depression may reduce the incidence of joint revision,^79^ and further investigation into medication regimes would be beneficial. Regardless, theme 4 clearly illustrated from the patient's perspective that care after TKR is suboptimal, and more individualised treatment is needed. Further work within this space is clearly warranted.

Our study has several strengths, including a preregistered protocol, an extensive and systematic search strategy, use of independent study screening and inclusion procedures (whereby researchers were blinded to the other's decisions) and reporting consistent with gold standard recommendations (eg, ENTREQ). Furthermore, use of an iterative data analysis process, undertaken by multiple authors and incorporating continued reflexivity reduces the risk of biased interpretation. There are also limitations. Despite an extensive search, some studies were excluded due to insufficient reporting (ie, unable to confirm eligibility); thus, it is possible that some relevant perspectives were not fully explored. Furthermore, the generalizability of this meta-synthesis is primarily limited to perspectives of western developed nations and to a population of people older than 60 years undergoing TKR. It is possible that additional or different issues may be relevant for younger people undergoing TKR and in developing nations.

## 5. Conclusions

This meta-synthesis provides key perspectives from patients undergoing TKR that highlight the importance of better information about the surgery and what to expect both postsurgical and longer term, including the scope of ‘normal’ recovery trajectories. The varied recovery trajectories identified here will provide a key resource for patients and clinicians. Our results also bring to light the lack of available resources and support for people post-TKR, particularly services that are individualised to the patient's unique context. While many patients will have a good clinical outcome after TKR surgery, there is a clear gap in present care that leaves some patients fearful, worried, anxious, and discontented, with negative effects on life participation. The meta-synthesis highlights that the future advances and improvements in clinical outcome after TKR will likely come from targeting the patient experience, including expectations, knowledge, and support, rather than from improvements in the surgical intervention itself.

## Disclosures

Carrie E. V. Taylor receives payment for providing courses on pain and well-being, and for providing general health-related workshops. Tasha R. Stanton receives speaker fees for lectures on pain and rehabilitation and receives royalties from NOIGroup Pty Ltd for a book published on Osteoarthritis Modern Pain Science.. Carolyn M. Murray has no conflicts to declare.

## Appendix A. Supplemental digital content

Supplemental digital content associated with this article can be found online at http://links.lww.com/PR9/A159.

## Supplementary Material
