# Supplementary material for: Patient perspectives of pain and function after knee replacement: a systematic review and meta-synthesis of qualitative studies
Source: Pain Rep. 2022 May 9;7(3):e1006. doi: 10.1097/PR9.0000000000001006 (PMC9088230; doi:10.1097/PR9.0000000000001006)
Supplement: Supplementary file 1 [file painreports-7-e1006-s001.pdf]

Supplementary File 1: Enhancing transparency in reporting the synthesis of qualitative research: ENTREQ checklist (Tong et al, 2012)

#	Item	Description	Location
1	Aim	State the research question the synthesis addresses	Introduction
2	Synthesis methodology	Identify the synthesis methodology or theoretical framework which underpins the synthesis, and describe the rationale for choice of methodology (e.g. meta-ethnography, thematic synthesis, critical interpretive synthesis, grounded theory synthesis, realist synthesis, meta-aggregation, meta-study, framework synthesis)	Introduction and Method
3	Approaching to searching	Indicate whether the search was pre-planned (comprehensive search strategies to seek all available studies) or iterative (to seek all available concepts until they theoretical saturation is achieved)	Study registration and reporting, Data sources and search strategy
4	Inclusion criteria	Specify the inclusion/exclusion criteria (e.g. in terms of population, language, year limits, type of publication, study type)	Eligibility criteria and study selection
5	Data sources	Describe the information sources used (e.g. electronic databases (MEDLINE, EMBASE, CINAHL, psycINFO), grey literature databases (digital thesis, policy reports), relevant organisational websites, experts, information specialists, generic web searches (Google Scholar) hand searching, reference lists) and when the searches conducted; provide the rationale for using the data sources	Data sources and search strategy
6	Electronic search strategy	Describe the literature search (e.g. provide electronic search strategies with population terms, clinical or health topic terms, experiential or social phenomena related terms, filters for qualitative research, and search limits)	Data sources and search strategy, Supplementary file 1: Database specific search criteria
7	Study screening methods	Describe the process of study screening and sifting (e.g. title, abstract and full text review, number of independent reviewers who screened studies)	Data handling and study inclusion
8	Study characteristics	Present the characteristics of the included studies (e.g. year of publication, country, population, number of participants, data collection, methodology, analysis, research questions)	Results<br>Table 2: Study characteristics
9	Study selection results	Identify the number of studies screened and provide reasons for study exclusion (e.g. for comprehensive searching, provide numbers of studies	

		screened and reasons for exclusion indicated in a figure/flowchart; for iterative searching describe reasons for study exclusion and inclusion based on modifications to the research question and/or contribution to theory development)	
10	Rationale for appraisal	Describe the rationale and approach used to appraise the included studies or selected findings (e.g. assessment of conduct (validity and robustness), assessment of reporting (transparency), assessment of content and utility of the findings)	CASP table
11	Appraisal items	State the tools, frameworks and criteria used to appraise the studies or selected findings (e.g. Existing tools: CASP, QARI, COREQ, Mays and Pope [25]; reviewer developed tools; describe the domains assessed: research team, study design, data analysis and interpretations, reporting)	Quality assessment
12	Appraisal process	Indicate whether the appraisal was conducted independently by more than one reviewer and if consensus was required	Qualitative meta-synthesis outcomes
13	Appraisal results	Present results of the quality assessment and indicate which articles, if any, were weighted/excluded based on the assessment and give the rationale	Quality assessment
14	Data extraction	Indicate which sections of the primary studies were analysed and how were the data extracted from the primary studies? (e.g. all text under the headings "results /conclusions" were extracted electronically and entered into a computer software)	Data extraction
15	Software	State the computer software used, if any	None used
16	Number of reviewers	Identify who was involved in coding and analysis	
17	Coding	Describe the process for coding of data (e.g. line by line coding to search for concepts)	Data analysis
18	Study comparison	Describe how were comparisons made within and across studies (e.g. subsequent studies were coded into pre-existing concepts, and new concepts were created when deemed necessary)	
19	Derivation of themes	Explain whether the process of deriving the themes or constructs was inductive or deductive	
20	Quotations	Provide quotations from the primary studies to illustrate themes/constructs, and identify whether the quotations were participant quotations of the author's interpretation	Findings<br>Table 4: illustrative quotes

21	Synthesis output	Present rich, compelling and useful results that go beyond a summary of the primary studies (e.g. new interpretation, models of evidence, conceptual models, analytical framework, development of a new theory or construct)	Findings and Discussion

Supplementary File 2: Database specific search criteria

Databases:

EMBASE

1	Exp total knee arthroplasty/
2	("knee replacement" or "total knee replacement" or "unicondylar knee replacement" or "knee joint replacement" or "knee arthroplasty" or "TKA" or "TKR").mp. [mp=title, abstract, original title, name of substance word, subject heading word, floating sub-heading word, keyword heading word, organism supplementary concept word, protocol supplementary concept word, rare disease supplementary concept word, unique identifier, synonyms]
3	1 or 2
4	Qualitative research/
5	Telephone interview/ or interview/ or semi structured interview/
6	Personal experience/ or experience
7	Perception/
8	(perce* or dissatisf* or qualitative or interview or experien*or focus group).mp. [mp=title, abstract, original title, name of substance word, subject heading word, floating sub-heading word, keyword heading word, organism supplementary concept word, protocol supplementary concept word, rare disease supplementary concept word, unique identifier, synonyms]
9	4 or 5 or 6 or 7 or 8
10	Exp pain/
11	Function.mp
12	10 or 11
13	3 and 9 and 12

EMCARE

1	exp total knee replacement/ or exp knee replacement/ or exp knee arthroplasty/
2	("knee replacement" or "total knee replacement" or "knee joint replacement" or "unicondylar knee replacement" or "TKA" or "TKR").mp.
3	1 or 2
4	qualitative research/
5	semi structured interview/ or telephone interview/ or interview/ or structured interview/ or unstructured interview/
6	personal experience/ or experience/
7	perception/
8	(perce* or dissatisf* or qualitative or interview or experien* or focus group).mp. [mp=title, abstract, original title, name of substance word, subject heading word, floating sub-heading word, keyword heading word, organism supplementary concept word, protocol supplementary concept word, rare disease supplementary concept word, unique identifier, synonyms]
9	4 or 5 or 6 or 7 or 8
10	exp pain/
11	function.mp
12	10 or 11
13	3 and 9 and 12

Cochrane Database

#1	MeSH descriptor: [Arthroplasty, Replacement, Knee] this term only
#2	MeSH descriptor: [Qualitative Research] explode all trees
#3	MeSH descriptor: [Pain] explode all trees
#4	‘knee replacement’ OR ‘knee arthroplasty’ OR ‘total knee replacement’ OR ‘total knee arthroplasty’
#5	‘qualitative’ or ‘interview’ or ‘focus group’ or ‘perce*’ or ‘experien*’ or ‘dissatisf*’
#6	‘pain’ or ‘function’
#7	#1 or #4
#8	#2 or #5
#9	#3 or #6
#10	#7 and # 8 and #9

MEDLINE

1	Arthroplasty, Replacement, Knee/
2	("knee replacement" or "total knee replacement" or "knee joint replacement" or "unicondylar knee replacement" or "TKA ot TKR").mp. [mp=title, abstract, original title, name of substance word, subject heading word, floating sub-heading word, keyword heading word, organism supplementary concept word, protocol supplementary concept word, rare disease supplementary concept word, unique identifier, synonyms]
3	1 or 2
4	Qualitative research
5	interview
6	Perception/ or Pain Perception
7	Focus groups/
8	(qualitative or dissatisf* or perce* or interview* or experien* or "focus group").mp. [mp=title, abstract, original title, name of substance word, subject heading word, floating sub-heading word, keyword heading word, organism supplementary concept word, protocol supplementary concept word, rare disease supplementary concept word, unique identifier, synonyms]
9	4 or 5 or 6 or 7 or 8
10	exp Pain/
11	"Recovery of Function"/
12	(pain or function).mp. [mp=title, abstract, original title, name of substance word, subject heading word, floating sub-heading word, keyword heading word, organism supplementary concept word, protocol supplementary concept word, rare disease supplementary concept word, unique identifier, synonyms]
13	10 or 11 or 12
14	3 and 9 and 13

ProQuest Dissertations & Theses Global

	Total knee replacement or knee replacement or knee arthroplasty or TKR or TKA or unicondylar replacement	in Abstract
AND	Qualitative or interview or focus group or experien* or perce*or dissatisf*	in Abstract
AND	Pain or function	in Abstract

PsycINFO

1	("knee replacement" or "total knee replacement" or "knee joint replacement" or "knee arthroplasty" or "total knee arthroplasty" or "unicondylar" or " TKA" or "TKR").mp. [mp=title, abstract, heading word, table of contents, key concepts, original title, tests & measures, mesh]
2	Qualitative Methods/
3	exp interviews/
4	exp focus groups/
5	Perception/ or Pain Perception
6	(qualitative or perce* or interview* or "focus group" or experien*).mp. [mp=title, abstract, heading word, table of contents, key concepts, original title, tests & measures, mesh]
7	2 or 3 or 4 or 5 or 6
8	Exp Pain/
9	function.mp
10	8 or 9
11	1 and 7 and 10

Scopus

Search	"total knee replacement" or "total knee arthroplasty" or "knee replacement" or "knee arthroplasty" AND	Article title, Abstract, Keywords
Search	qualitative or perce*or experien* or interview* or focus group or dissatisfy* AND	Article title, Abstract, Keywords
Search	Pain or function	Article title, Abstract, Keywords

Websites:

Google Scholar

Keywords	"total knee replacement," "qualitative," "pain," "function."

Trove

Keywords	"total knee replacement" and "qualitative" and "pain" or "function"

Supplementary File 3: CASP Checklist and Critical Appraisal Findings

3a: CASP Checklist

	Aim	Purpose is Qualitative	Research design	Recruitment	Data collection	Researcher relationship	Ethics	Data analysis	Findings	TOTAL
Berg et al;2019	3	3	2	3	3	1	3	3	3	24
Bremner; 2011, 2012	3	3	3	3	3	1	3	3	3	25
Bunzli et al; 2020	3	3	2	3	3	3	3	2	3	26
Coutu et al; 2020	3	3	3	3	3	2	3	3	3	26
Engstrom et al; 2017	3	3	3	3	3	1	3	3	3	25
Fletcher et al; 2019	3	3	3	3	3	2	1	2	3	23
Harding et al; 2015	3	3	3	3	3	2	2	3	3	25
Jeffrey et al; 2011	3	3	2	2	2	1	2	3	3	21
Johnson et al; 2014	3	3	3	3	3	1	3	3	2	24
Kleiner et al; 2004	3	3	3	3	3	1	3	3	3	25
Klem et al; 2020	3	3	3	3	3	3	3	3	3	27
Loth et al; 2018	3	3	1	3	3	2	3	3	3	24
Mahdi et al; 2020	3	3	2	3	3	2	3	3	3	25
Maillette et al; 2017	3	3	2	3	3	2	3	3	3	25
Marcinkowski et al; 2005	3	3	3	3	2	2	3	3	3	25
Moore & Gooberman-Hill; 2020	3	3	2	3	3	2	3	2	3	24
Pellegrini et al; 2018	3	3	1	3	3	1	2	2	3	21
Perry et al; 2011	3	3	3	3	3	3	3	2	3	26
Sjoveian et al; 2017	3	3	3	3	3	2	2	2	3	24
Smith et al; 2020	3	3	2	3	3	1	3	3	3	24
Specht et al; 2018	3	3	3	2	3	3	3	3	3	26
Specht et al; 2016	3	3	3	2	3	3	3	3	3	26
Stenquist et al; 2015	3	3	2	2	3	1	2	3	3	22
Webster et al; 2015	3	3	3	2	3	3	2	3	3	25

Woolhead et al; 2005	3	3	2	2	2	1	3	3	2	21
Wylde et al; 2012	3	3	2	3	3	1	3	3	3	24
Zacharia et al; 2016	2	3	1	2	3	1	3	2	3	20

Refer to CASP tool for full description of criteria; Question 10 (Utility) not awarded a score. Possible minimum 9; possible maximum 27; Scores: 3 deemed to provide extensive justification and explanation of the criteria (highest score); 2 deemed to address the criteria but did not fully elaborate; 1 deemed to offer little to no justification of the criteria (lowest score).

3b: Critical Appraisal Findings

Paper	Critique
Berg et al;2019	Research design partly described. Participant researcher relationships not reported.
Bremner; 2011, 2012	Participant researcher relationships not reported.
Bunzli et al; 2020	Research design partly described. Data analysis partly described.
Coutu et al; 2020	Participant researcher relationships not fully reported
Engstrom et al; 2017	Participant researcher relationships not reported.
Fletcher et al; 2019	Participant researcher relationships not fully reported. Ethics minimally reported. Data analysis partly described.
Harding et al; 2015	Participant researcher relationships not fully reported. Ethics not fully reported.
Jeffrey et al; 2011	Participant recruitment not adequately described. Data collection partly described. Participant researcher relationships not reported. Ethics not fully reported.
Johnson et al; 2014	Participant researcher relationships not reported. Findings were not fully developed.
Kleiner et al; 2004	Participant researcher relationships not reported.
Klem et al; 2020	Criteria all well described.
Loth et al; 2018	Research design is incomplete. Participant researcher relationships not fully reported.
Mahdi et al; 2020	Research design partly described. Participant researcher relationships not fully reported.
Maillette et al; 2017	Research design partly described. Participant researcher relationships not fully reported.
Marcinkowski et al; 2005	Data collection partly described. Participant researcher relationships not fully reported.
Moore & Gooberman-Hill; 2020	Research design partly described. Participant researcher relationships not fully reported. Data analysis partly described.
Pellegrini et al; 2018	Research design incomplete. Participant researcher relationships not reported. Ethics not fully reported. Data analysis partly described.
Perry et al; 2011	Participant researcher relationships not fully reported. Data analysis partly described.
Sjoveian et al; 2017	Participant researcher relationships not fully reported. Ethics not fully reported. Data analysis partly described.
Smith et al; 2020	Research design is incomplete. Participant researcher relationships not reported.
Specht et al; 2018	Participant recruitment not adequately described.
Specht et al; 2016	As above
Stenquist et al; 2015	Research design partly described. Participant recruitment not adequately described. Participant researcher relationships not reported. Ethics not fully reported.
Webster et al; 2015	Participant recruitment not adequately described. Ethics not fully reported.

Woolhead et al; 2005	Research design partly described. Participant recruitment not adequately described. Data collection partly described. Participant researcher relationships not reported. Findings were not fully developed.
Wylde et al; 2012	Research design partly described. Participant researcher relationships not reported.
Zacharia et al; 2016	Research aim needs more clarification. Research design incomplete. Participant recruitment not adequately described. Participant researcher relationships not reported. Data analysis partly described.

Supplementary file 4: List of all studies included in the meta-synthesis

List of all included studies
Berg U, Berg M, Rolfson O, Erichsen-Andersson A. Fast-track program of elective joint replacement in hip and knee-patients' experiences of the clinical pathway and care process. J Orthop Surg Res 2019;14(1):186-186.
Bremner S. A Qualitative Descriptive Study: Older Adults' Postoperative Pain Medication Usage After Total Knee Arthroplasty. In: C McCartney, F Webster editors: ProQuest Dissertations Publishing, 2011.
Bremner S, Webster, F., Katz, J., Watt-Watson, J., McCartney, C. Older adults' postoperative pain medication usage after total knee arthroplasty: A qualitative descriptive study. Journal of Opioid Management 2012;8(3):145.
Bunzli S, O'Brien P, Klem N, Incoll I, Singh J, Davaris M, Choong P, Dowsey M. Misconceived expectations: Patient reflections on the total knee replacement journey. Musculoskeletal Care 2020;18(4):415-424.
Coutu MF, Gaudreault N, Major ME, Nastasia I, Dumais R, Deshaies A, Pettigrew S, Labrecque ME, Desmeules F, Maillette P. Return to work following total knee arthroplasty: A multiple case study of stakeholder perspectives. Clinical Rehabilitation 2020.
Engström Å, Boström J, Karlsson A-C. Women's Experiences of Undergoing Total Knee Joint Replacement Surgery. J Perianesth Nurs 2017;32(2):86-95.
Fletcher D, Moore AJ, Blom AW, Wylde V. An exploratory study of the long-term impact of difficulty kneeling after total knee replacement. Disabil Rehabil 2019;41(7):820-825.
Harding PA, Holland AE, Hinman RS, Delany C. Physical activity perceptions and beliefs following total hip and knee arthroplasty: a qualitative study. Physiother Theory Pract 2015;31(2):107-113.
Jeffery AE, Wylde V, Blom AW, Horwood JP. “It's there and I'm stuck with it”: Patients' experiences of chronic pain following total knee replacement surgery. Arthritis Care Res (Hoboken) 2011;63(2):286-292.
Johnson EC, Horwood J, Gooberman-Hill R. Patients’ Journeys through Total Joint Replacement: Patterns of Medication Use. Musculoskeletal Care 2014;12(2):92-102.
Kleiner C. Experiences of pain in elderly patients having total knee arthroplasty. Ann Arbor: Duquesne University, 2004. p. 122.
Klem NR, Smith A, O'Sullivan P, Dowsey MM, Schutze R, Kent P, Choong PF, Bunzli S. What influences patient satisfaction after TKA? A qualitative investigation. Clinical Orthopaedics and Related Research 2020;478(8):1850-1866.
Loth FL, Liebensteiner MC, Giesinger JM, Giesinger K, Bliem HR, Holzner B. What makes patients aware of their artificial knee joint? BMC Musculoskelet Disord 2018;19(1):5-5.
Mahdi A, Svantesson M, Wretenberg P, Halleberg-Nyman M. Patients' experiences of discontentment one year after total knee arthroplasty- a qualitative study. BMC Musculoskelet Disord 2020;21(1):29.
Maillette P, Coutu MF, Gaudreault N. Workers' perspectives on return to work after total knee arthroplasty. Annals of Physical and Rehabilitation Medicine 2017;60(5):299-305.
Marcinkowski K, Wong VG, Dignam D. Getting back to the future: a grounded theory study of the patient perspective of total knee joint arthroplasty. Orthopedic nursing 2005;24(3):202-209.
Moore AJ, Gooberman-Hill R. Why don't patients seek help for chronic post-surgical pain after knee replacement? A qualitative investigation. Health Expectations 2020.
Pellegrini CA, Ledford G, Chang RW, Cameron KA. Understanding barriers and facilitators to healthy eating and physical activity from patients either before and after knee arthroplasty. Disability and Rehabilitation 2018;40(17):2004-2010.
Perry MAC, Hudson S, Ardis K. "If I didn't have anybody, what would I have done?": Experiences of older adults and their discharge home after lower limb orthopaedic surgery. Journal of rehabilitation medicine 2011;43(10):916-922.
Sjoveian AKH, Leegaard M. Hip and knee arthroplasty - patient's experiences of pain and rehabilitation after discharge from hospital. International journal of orthopaedic and trauma nursing 2017;27:28-35.
Smith S, Alvand A, Locock L, Ryan S, Smith J, Bayliss L, Wilson H, Price A. Partial or total knee replacement? Identifying patients' information needs on knee replacement surgery: a qualitative study to inform a decision aid. Quality of Life Research 2020;29(4):999-1011.

Specht K, Agerskov H, Kjaersgaard-Andersen P, Jester R, Pedersen BD. Patients' experiences during the first 12 weeks after discharge in fast-track hip and knee arthroplasty - a qualitative study. International journal of orthopaedic and trauma nursing 2018;31:13-19.
Specht K, Kjaersgaard-Andersen P, Pedersen BD. Patient experience in fast-track hip and knee arthroplasty--a qualitative study. Journal of clinical nursing 2016;25(5-6):836-845.
Stenquist DS, Elman SA, Davis AM, Bogart LM, Brownlee SA, Sanchez ES, Santiago A, Ghazinouri R, Katz JN. Physical activity and experience of total knee replacement in patients one to four years postsurgery in the Dominican Republic: A qualitative study. Arthritis Care and Research 2015;67(1):65-73
Webster F, Perruccio AV, Jenkinson R, Jaglal S, Schemitsch E, Waddell JP, Venkataramanan V, Bytautas J, Davis AM. Understanding why people do or do not engage in activities following total joint replacement: a longitudinal qualitative study. Osteoarthritis and Cartilage 2015;23(6):860-867.
Woolhead GM, Donovan JL, Dieppe PA. Outcomes of total knee replacement: A qualitative study. Rheumatology 2005;44(8):1032-1037.
Wylde V, Jeffery A, Dieppe P, Gooberman-Hill R. The assessment of persistent pain after joint replacement. Osteoarthritis and Cartilage 2012;20(2):102-105.
Zacharia B, Paul M, Thanveeruddin Sherule M. Patient-based outcome analysis is important to determine the success of total knee arthroplasty: result of a focus group discussion. Medical devices (Auckland, NZ) 2016;9:125-130.
